# Analgesic Effect of 5-(3,4-Dihydroxyphenyl)-3-hydroxy-1-(2-hydroxyphenyl)penta-2,4-dien-1-one in Experimental Animal Models of Nociception

**DOI:** 10.3390/molecules23092099

**Published:** 2018-08-21

**Authors:** Nadhirah Kamarudin, Nadia Hisamuddin, Hui Ming Ong, Ahmad Farhan Ahmad Azmi, Sze Wei Leong, Faridah Abas, Mohd Roslan Sulaiman, Wan Mastura Shaik Mossadeq

**Affiliations:** 1 Department of Veterinary Preclinical Sciences, Faculty of Veterinary Medicine, Universiti Putra Malaysia, Serdang 43400, Selangor, Malaysia; nurnadhirahkamarudin@gmail.com (N.K.); nadiahisamuddinn@gmail.com (N.H.); 2 Department of Biomedical Sciences, Faculty of Medicine and Health Sciences, Universiti Putra Malaysia, Serdang 43400, Selangor, Malaysia; oguiming85@gmail.com (H.M.O.); farhanazmirahman@gmail.com (A.F.A.A.); mrs@upm.edu.my (M.R.S.); 3 Department of Microbiology, Faculty of Biotechnology and Biomolecular Sciences, Universiti Putra Malaysia, Serdang 43400, Selangor, Malaysia; leongszewei@upm.edu.my; 4 Laboratory of Natural Products, Institute of Bioscience, Universiti Putra Malaysia, Serdang 43400, Selangor, Malaysia; faridah_abas@upm.edu.my; 5 Department of Food Science, Faculty of Food Science and Technology, Universiti Putra Malaysia, Serdang 43400, Selangor, Malaysia

**Keywords:** pain, 5-(3,4-dihydroxyphenyl)-3-hydroxy-1-(2-hydroxyphenyl)penta-2,4-dien-1-one, turmeric, antinociceptive, vanilloid, glutamatergic, opioid

## Abstract

Curcuminoids derived from turmeric rhizome have been reported to exhibit antinociceptive, antioxidant and anti-inflammatory activities. We evaluated the peripheral and central antinociceptive activities of 5-(3,4-dihydroxyphenyl)-3-hydroxy-1-(2-hydroxyphenyl)penta-2,4-dien-1-one (**DHHPD**), a novel synthetic curcuminoid analogue at 0.1, 0.3, 1 and 3 mg/kg (intraperitoneal), through chemical and thermal models of nociception. The effects of **DHHPD** on the vanilloid and glutamatergic systems were evaluated through the capsaicin- and glutamate-induced paw licking tests. Results showed that **DHHPD** significantly (*p* < 0.05) attenuated the writhing response produced by the 0.8% acetic acid injection. In addition, 1 and 3 mg/kg of **DHHPD** significantly (*p* < 0.05) reduced the licking time spent by each mouse in both phases of the 2.5% formalin test and increased the response latency of mice on the hot-plate. However, the effect produced in the latter was not reversed by naloxone, a non-selective opioid receptor antagonist. Despite this, **DHHPD** decreased the licking latency of mice in the capsaicin- and glutamate-induced paw licking tests in a dose response manner. In conclusion, **DHHPD** showed excellent peripheral and central antinociceptive activities possibly by attenuation of the synthesis and/or release of pro-inflammatory mediators in addition to modulation of the vanilloid and glutamatergic systems without an apparent effect on the opioidergic system.

## 1. Introduction

Pain decreases the quality of a person’s life and results in expensive medical expenses and economic losses to the society [1]. The bodily and emotional experience related to this condition is often associated with potential or actual tissue damage as a consequence of exposure to intense or damaging stimuli [2].

Pain management primarily rely on anti-inflammatory and antinociceptive medications such as opiates and non-steroidal anti-inflammatory drugs (NSAIDs). Drugs that are prescribed for the management or control of pain associated with inflammatory conditions are usually antagonists of endogenous pro-inflammatory mediators such as prostaglandins, leukotrienes and histamine. These drugs target the H1 receptor for histamine, cyclooxygenases 1 and 2, tumour necrosis factor-α and cycteinyl leukotrienes C4 and D4 receptors, to name a few [3]. Numerous animal studies dated as far back as in the 1970s and 1980s indicated that the opening of specific K^+^ channels by agonists of many G-protein coupled receptors (e.g., serotonin 5-HT1A, adenosine A1, α2-adrenoreceptors, muscarinic M2, GABA, opioid and cannabinoid receptors), tricyclic antidepressants, NSAIDs and natural products, have all induced antinociceptive activity in the acute and chronic models of pain [4]. It then led scientists to hypothesize that blockade of these channels by pro-inflammatory mediators and specific channel blockers may produce increased hyperalgesia during inflammation. However, the prolonged use of these drugs is often accompanied with side effects such as gastrointestinal ulcer [5], respiratory depression [1], high risk of stroke [6] and haemorrhage [7].

Narcotic agents such as morphine and codeine or synthetic derivatives possessing morphine-like properties such as heroin are potent analgesics and are frequently used for relief of severe pain in spite of their potential for abuse [8]. This group of drugs inhibit nociception by binding to the opioid receptors on the primary afferent as well as neurons in the dorsal horn of the spinal cord and in the brain, consequently exacerbating the descending inhibitory effect of nociceptive signalling at this level [9,10]. However, these drugs induce central and peripheral adverse side effects including drowsiness, sedation, cough suppression, vomiting, pupillary constriction, hypotension, withdrawal symptoms, pruritus and delirium [11,12,13,14]. Addicts and patients undergoing opiate-based treatments often succumb to constipation, resulting from opiate-induced cholinergic activity stimulation in the gut wall ganglia, in addition to bronchospasm, flushing and arteriolar dilatation, as a result of histamine release. Often given to patients who are undergoing treatment for chronic or long term debilitating diseases, prescriptions of narcotics in the treatment of pain have been generating a lot of controversies since use of narcotics were usually associated with tolerance and addiction in susceptible patients [15]. However, addiction to opioids, if present, is to an extent, dependent on the individual’s exposure to drugs, genetic, social and psychological factors [16,17,18,19].

Hence, the search for an alternative to these medications such as naturally-occurring products is gaining attention among researchers and public in hope that these natural substances may induce desired outcomes similar to existing medication but with lesser or zero side effects.

Rhizomes have been a popular choice of remedy for various common ailments in folklore medicine due to its abundance, all year availability, low cost and effectiveness. Aside from its medicinal use, several species of rhizomes such as ginger, fingerroot, and turmeric are often used as spice in soups and dishes in the Southeast Asia region.

*Zingiber zerumbet* (pinecone) for example, are traditionally used by the Indians, Malays, Chinese and Hawaiians to treat inflammation, fever, wound and bruises [20]. In addition, extracts of *Curcuma longa* (turmeric, *C. longa*) have been demonstrated to be effective against cancer, cough, diabetic wounds, dermatitis, rheumatism, sinusitis, high cholesterol and AIDS [21,22,23,24,25]. Commonly known as ‘kunyit’ in Malaysia, this plant is native to tropical countries such as Malaysia, India and China.

Curcuminoids, the bioactive components of *C. longa*, exhibit antioxidant, anti-inflammatory and antinociceptive activities [26]. Despite the benefits, curcuminoids have poor bioavailability and pharmacokinetic properties [27] thus reducing their potential as alternative medicinal and therapeutic agents. Hence, synthesis of curcuminoid analogues with good bioavailability, solubility and biological properties is warranted.

**Figure 1:**
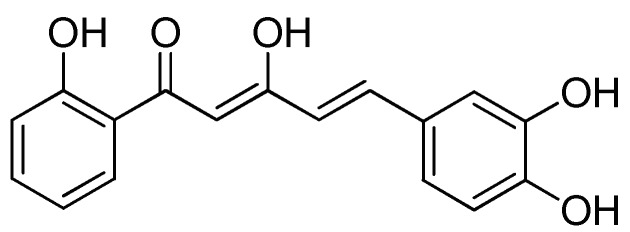
Chemical structure of 5-(3,4-dihydroxyphenyl)-3-hydroxy-1-(2-hydroxyphenyl)penta-2,4-dien-1-one (**DHHPD**).

The 5-(3,4-dihydroxyphenyl)-3-hydroxy-1-(2-hydroxyphenyl)penta-2,4-dien-1-one, or **DHHPD** (Figure 1) is a derivative of diarylpentanoids, an analogue of curcumin. Diarylpentanoids showed better bioavailability and greater pharmacological activities compared to curcumin as it showed significant anti-inflammatory properties in vitro [28,29]. Hence, the present study aims to evaluate the central and peripheral antinociceptive activities of **DHHPD** through chemical and thermal experimental animal models of nociception.

## 2. Results

### 2.1. Acute Toxicity Study

During the 7-day observation period, there were no abnormal behaviours, morbidity or mortality recorded. In addition, no abnormal changes were observed on the vital organs of mice during post-mortem.

### 2.2. Antinociceptive Studies

#### 2.2.1. Acetic Acid-Induced Writhing Test

**Figure 2:**
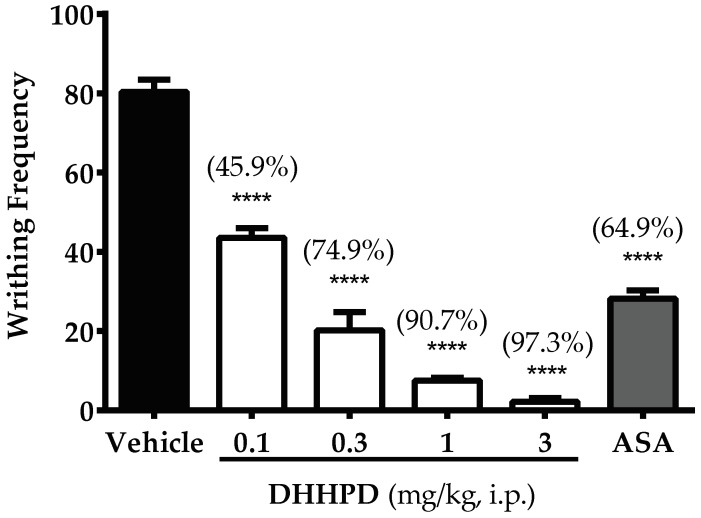
Effect of **DHHPD** against 0.8% acetic acid-induced abdominal constriction test in mice. Each column denotes the mean ± SEM (*n* = 6). The asterisks (*) represent significance level as compared to the vehicle group, **** *p* < 0.0001 by one-way ANOVA followed by Dunnett’s post hoc test. Mice were administered with vehicle (10 mL/kg, i.p.), acetylsalicylic acid (ASA, 100 mg/kg, i.p.) and **DHHPD** (0.1, 0.3, 1 and 3 mg/kg, i.p.).

Figure 2 illustrates the effect of **DHHPD** against 0.8% acetic acid-induced abdominal constriction/writhing test. The calculated mean ED_50_ of **DHHPD** for this test was 0.28 mg/kg, i.p. (CI, 0.17–0.48 mg/kg). **DHHPD** at the doses of 0.1, 0.3, 1 and 3 mg/kg exhibited significant (*p* < 0.05) reduction in writhing frequency by 45.9%, 74.9%, 90.7% and 97.3%, respectively. Similarly, the aspirin group (ASA) inhibited writhing by 64.9% as compared to the vehicle group.

#### 2.2.2. Formalin-Induced Paw Licking Test

**Figure 3:**
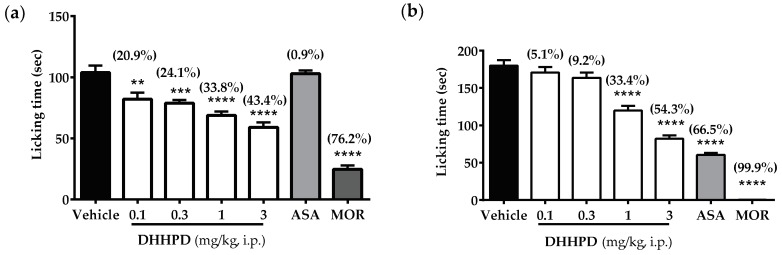
Effect of **DHHPD** against 2.5% formalin-induced paw licking test in mice. (**a**) Early/neurogenic phase; (**b**) Late/inflammatory phase. Each column represents the mean ± SEM (*n* = 6). The asterisks (*) denote the significance level as compared to the vehicle group, ** *p* < 0.01, *** *p* < 0.001, **** *p* < 0.0001 by one-way ANOVA followed by Dunnett’s post hoc test. Mice were administered with vehicle (10 mL/kg, i.p.), acetylsalicylic acid (ASA, 100 mg/kg, i.p.) and **DHHPD** (0.1, 0.3, 1 and 3 mg/kg, i.p.).

**DHHPD** at the doses of 0.1, 0.3, 1 and 3 mg/kg showed significant reduction in the latency/time that each mouse spent on biting and licking the injected hind paw (sec) during the neurogenic phase by 20.9%, 24.1%, 33.8% and 43.3%, respectively (Figure 3a). As expected, morphine produced the highest reduction of the licking time (76.2%) while ASA produced only a 0.9% decrease compared to vehicle.

In the late phase (Figure 3b), mice treated with **DHHPD** (3 mg/kg, i.p.) recorded significant reduction in licking time by 54.3% compared to mice treated with **DHHPD** at doses of 0.1, 0.3 and 1 mg/kg, with 5.1%, 9.2%, and 33.4% reduction, respectively. Likewise, the ASA and morphine groups showed 66.5% and 99.9% inhibition, respectively. The calculated mean ED_50_ of **DHHPD** for the early and late phases were 0.82 mg/kg, i.p. (CI, 0.54–1.3 mg/kg) and 0.87 mg/kg, i.p. (CI, 0.69–1.1 mg/kg), respectively. However, **DHHPD** at all doses reduced the latency of licking compared to the vehicle group. Compared to other doses, **DHHPD** at 1 and 3 mg/kg significantly (*p* < 0.05) reduced the nocifensive behaviour in both phases of the test.

#### 2.2.3. Hot-Plate Test

Intraperitoneal administration of **DHHPD** prolonged the response latency in mice at different time intervals, as shown in Table 1. A significant (*p* < 0.05) increase in response latency was observed beginning at 60 min and continued until 90 min, for all doses of **DHHPD**. The response latency for mice which received 0.1 and 0.3 mg/kg **DHHPD** gradually tapered off from this point until the end of the experiment. However, a continuous increase in latency was observed in mice which received **DHHPD** at 1 and 3 mg/kg until 150 min. Regardless, mice which received morphine (5 mg/kg, s.c.) showed the highest response latency compared to other treatment groups at all intervals until the end of the experiment. In addition, naloxone significantly antagonised analgesia produced by morphine at all intervals of the experiment.

#### 2.2.4. Involvement of the Opioidergic System

Naloxone (5 mg/kg, i.p.) did not antagonize the antinociceptive activity produced by the highest dose of **DHHPD** (3 mg/kg, i.p.) at all intervals as shown in Table 1.

#### 2.2.5. Capsaicin-Induced Paw Licking Test

**Figure 4:**
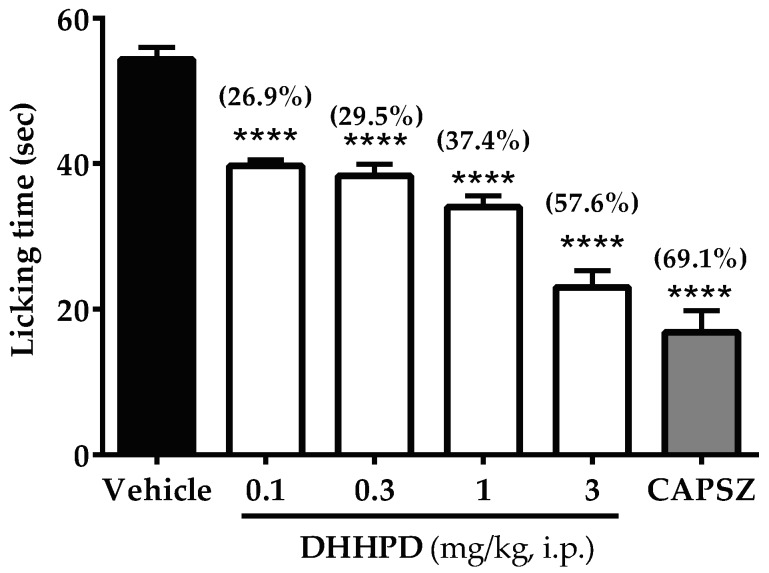
Effect of **DHHPD** against capsaicin-induced paw licking test in mice. Each column denotes the mean ± SEM (*n* = 6). The asterisks (*) represent the significance level as compared to the vehicle group, **** *p* < 0.0001 by one-way ANOVA followed by Dunnett’s post hoc test. Mice were administered with vehicle (10 mL/kg, i.p.), acetylsalicylic acid (ASA, 100 mg/kg, i.p.) and **DHHPD** (0.1, 0.3, 1 and 3 mg/kg, i.p.).

Figure 4 depicts the effect of **DHHPD** against capsaicin-induced paw licking test in mice. There were significant (*p* < 0.05) reductions in the licking time of mice treated with **DHHPD** at the doses of 0.1, 0.3, 1 and 3 mg/kg by 26.9%, 29.5%, 37.4% and 57.6%, respectively. However, the highest reduction was produced by capsazepine (69.1%). The calculated mean of ED_50_ for **DHHPD** in this test was 1.2 mg/kg, i.p. (CI, 0.67–2.0 mg/kg).

#### 2.2.6. Glutamate-Induced Paw Licking Test

**Figure 5:**
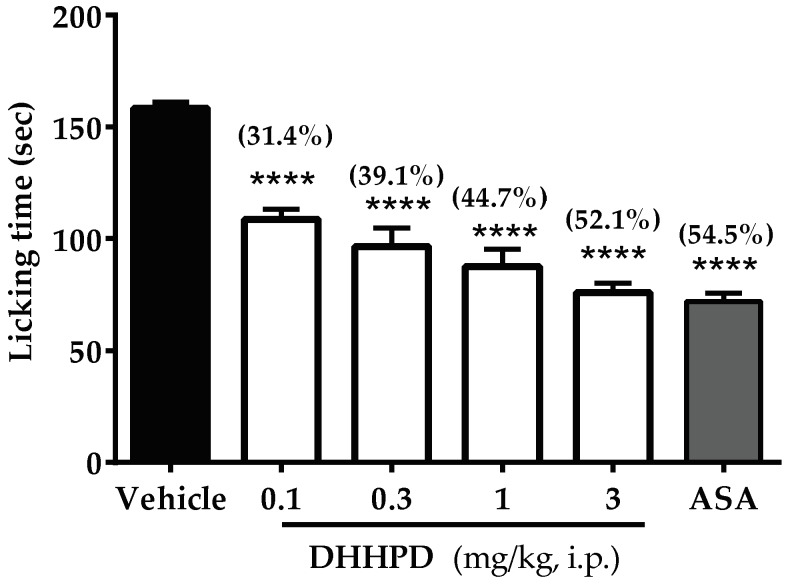
Effect of **DHHPD** against glutamate-induced paw licking test in mice. Each column represents the mean ± SEM (*n* = 6). The asterisks (*) denote the significance level as compared to the vehicle group, **** *p* < 0.0001 by one-way ANOVA followed by Dunnett’s post hoc test. Mice were pre-treated with vehicle (10 mL/kg, i.p.), acetylsalicylic acid (ASA, 100 mg/kg, i.p.) and **DHHPD** (0.1, 0.3, 1 and 3 mg/kg, i.p.).

The effect of **DHHPD** on the glutamate-induced paw licking test is shown in Figure 5. All doses of **DHHPD** (0.1, 0.3, 1 and 3 mg/kg) produced significant (*p* < 0.05) reductions of licking time by 31.4%, 39.1%, 44.7% and 52.1%, respectively. ASA produced an inhibition of 54.5%, similar to the result produced by the highest dose of **DHHPD**. The calculated mean ED_50_ of **DHHPD** was 0.54 mg/kg, i.p. (CI, 0.2–1.4 mg/kg).

### 2.3. Motor Performance Test

#### Rotarod Test

**Figure 6:**
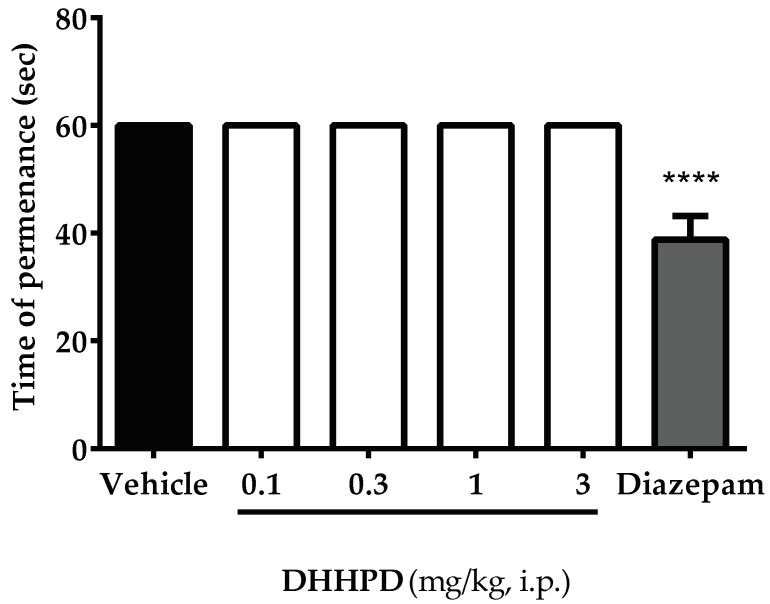
Effect of **DHHPD** on the rotarod test in mice. Each column denotes the mean ± SEM (*n* = 6). The asterisks (*) represent the significance level as compared to the vehicle group, **** *p* < 0.0001 by one-way ANOVA followed by Dunnett’s post hoc test. Mice were pre-treated with vehicle (10 mL/kg, i.p.), diazepam (4 mg/kg, i.p.) and **DHHPD** (0.1, 0.3, 1 and 3 mg/kg, i.p.).

The effect of **DHHPD** on rotarod test is shown in Figure 6. **DHHPD** at doses of 0.1, 0.3, 1 and 3 mg/kg (i.p.) did not change the motor performance of mice compared to the vehicle group. In contrast, mice which received diazepam (4 mg/kg, i.p.) demonstrated significant (*p* < 0.05) alterations in the motor performance or permanence on the rotarod. 

## 3. Discussion

The acetic acid-induced abdominal constriction test is one of the most sensitive tests to evaluate the peripheral analgesic activities of novel drugs, compound, derivatives or synthetic substances [30,31]. However, the effects produced are usually deemed non-specific, as the writhing response could be attenuated by antihistamines, adrenergic blockers and muscle relaxants [32,33]. Despite its non-specificity, this chemical model of nociception offers an immediate result on the analgesic properties of the aforementioned substances and products at the peripheral level. For instance, an intraperitoneal administration of acetic acid sensitizes the peripheral nociceptors producing pain as a result. The signals produced as a consequence to this event are transmitted to the central nervous system which will trigger the liberation of pro-inflammatory mediators such as histamine, bradykinin, serotonin, prostaglandins, substance P and endogenous enzymes such as lipoxygenase (LOX) and cyclooxygenase (COX-1 and COX-2) into the peritoneal fluid [30,31]. This series of events further sensitizes the nociceptors and the cycle is repeated until the algogenic agent or irritant is removed from the system or site of injury. NSAIDs which are often used in pain management work by attenuating the synthesis and/or release of pro-inflammatory mediators such as prostaglandins or enzymes which play a key role in inflammation such as LOX and COX. Drugs, natural product compounds or derivatives that induce antinociceptive activity in the acetic acid-induced abdominal constriction test are said to resemble the NSAIDs’ mechanism of action and are often considered as a promising alternative to peripherally-acting drugs [31].

Our present data showed that **DHHPD** at all doses significantly reduced the frequency of writhing comparably to ASA. This indicates that DHPPD may resemble NSAIDs in view of its mechanism of action in inducing analgesia. As the results of this test may be reproduced by other muscle relaxants or sedative agents, the rotarod test is required to rule out this possibility. Our data showed that mice were able to maintain their permanence on the rotarod, implying that the administration of **DHHPD** did not induce any sedative or muscle relaxing activity at all doses tested. In addition to the writhing test, highly specific tests such as the formalin-induced paw licking and hot-plate tests are recommended to verify the results obtained [34].

The 2.5% and 5% formalin-induced paw-licking tests are used to validate the peripheral and central antinociceptive effects of any novel drug, compound or substances. This test produces a distinct biphasic nociceptive response commonly referred to as the neurogenic/early phase and inflammatory /late phase [35].

An intraplantar injection of formalin on a mouse hind paw promotes direct activation of nociceptive neurons through the involvement of the C-fibres [36,37]. Consequently, neuropeptides such as substance P and bradykinin are released [38,39,40]. Pain produced during 0–5 min post formalin injection (neurogenic phase) may be mediated by the activation of transient receptor potential vanilloid 1 (TRPV1) [30]. During this phase, NSAIDs which specifically inhibit COX activity remain less effective in attenuating the nocifensive behaviour exhibited by mice compared to centrally-acting drugs such as morphine [41]. Our present results showed that ASA did not inhibit the licking time of mice in the formalin test during the early phase which confirms the previous findings. Unlike ASA, **DHHPD** at 0.1, 0.3, 1 and 3 mg/kg reduced the latency of licking during this phase but the effects produced by **DHHPD** at all doses were not as effective as morphine.

The late phase or inflammatory phase involves the release of several inflammatory mediators [31] such as histamine, serotonin, bradykinin, prostaglandin and excitatory amino acids as the tissue progressively undergo damage [42]. Similar to ASA, **DHHPD** at 1 and 3 mg/kg significantly reduced the latency of licking in this phase indicating that it may reduce and/or inhibit the synthesis or release of these mediators or is partly involved in modulating the transduction pathways in the control of pain at the peripheral level and hence, reduces the painful sensation in mice. Moreover, **DHHPD** at these doses significantly reduced the licking time in not only one but both phases of the formalin test. It is suggested that centrally-acting drugs such as opiates inhibit the nocifensive behaviour produced in both phases of the test whereas peripherally-acting drugs inhibit abnormal behaviour only during the late phase [43]. Thus, **DHHPD** at 1 and 3 mg/kg may resemble a centrally-acting drug in inducing analgesia, although the same is untrue for lower doses of **DHHPD**. Thus, further assessment such as the hot-plate test that is considered as a more specific test for the screening of centrally-acting drugs is recommended for confirmation. Regardless, the reduction of licking behaviour by 1 and 3 mg/kg **DHHPD** observed during the late phase of the 2.5% formalin test corresponds to effect produced by the same doses in the acetic-acid writhing test, confirming an involvement of peripherally mediated analgesia.

The hot-plate test is a thermally-induced pain model that evokes a spinal reflex or behavioural reaction and is often used to assess extracts or compounds that produces supra-spinal analgesia [30,34]. The present results showed that **DHHPD** at 1 and 3 mg/kg significantly prolonged the response latency in mice, confirming centrally-acting activities which correspond well with the results obtained from the formalin test. 

The hot-plate test can also be used to evaluate the involvement of opioid-mediated mechanism [44]. This mechanism of action involves the activation of either the mu (µ), kappa (ĸ) or delta (δ) opioid receptors [45]. Furthermore, the involvement of opioid receptor in compound- or substance-induced analgesia can be investigated through the pre-treatment of mice with naloxone, a non-selective opioid receptor antagonist [46]. A significant reversal of the antinociceptive effect produced suggests that the effects observed may involve an opioid-mediated mechanism [44]. However, our results showed that naloxone did not reverse the antinociceptive response produced by **DHHPD** indicating that the central analgesic activity of **DHHPD** was not mediated through the opioidergic system. Despite this, a prolongation of response latency by the highest doses of **DHHPD** in this test confirms the central activity exhibited by these doses during the early/neurogenic phase of the 2.5% formalin test.

We determined the involvement of Transient Receptor Potential Vanilloid 1 (TRPV1) in **DHHPD**-induced analgesia. This ligand-gated non-selective channel can be activated by high temperature (>43 °C), protons, and capsaicin, a phenolic compound found in the hot chilli peppers which is responsible for its burning and irritant effect [47,48]. An intraplantar injection of capsaicin (1.6 µg/paw, 20 µL) for instance, induces nocifensive responses such as licking and biting of the injected paw. Capsazepine, a standard reference drug for this test, has been shown to potently block TRPV1 receptor to induce analgesia and hence, reduces duration of licking and biting of the affected paw. In the present study, the administration of **DHHPD** (0.1, 0.3, 1 and 3 mg/kg, i.p.) inhibited the nocifensive response in the capsaicin-induced paw licking test, suggesting the inactivation of TRPV1 receptors in the induction of analgesia. Our result is in accordance to [47,49,50], whereby curcumin antagonizes the TRPV1 receptors by reducing the rise in calcium induced by capsaicin administration and inward flow of current in the dorsal root ganglion neurons in both rats and mice. The detailed effects of **DHHPD** on calcium flow and higher centres require further investigation. 

Glutamate receptors also play a crucial role in the pain pathway that modulates peripheral, spinal and supra-spinal analgesia [51]. This test evoked paw-licking and paw-biting behaviours. An intraplantar injection of glutamate solution activates the glutamatergic system via its specific ionotropic glutamate receptors (iGluRs), AMPA (α-amino-3-hydroxy-5-methyl-d-aspartate), NMDA (*N*-methyl-d-aspartate), kainate (KA) and metabotropic glutamate receptors (mGluRs) [52,53]. This system may also be mediated by the release of nitric oxide (NO) or other NO-derived substances [40]. The release of NO further increases the production of pro-inflammatory mediators such as cytokines, reactive oxygen species (ROS) and prostanoids [54,55]. **DHHPD** has in fact, been shown to suppress NO in vivo [28]. Furthermore, administration of **DHHPD** (0.1, 0.3, 1 and 3 mg/kg, i.p.) significantly attenuated the glutamate-induced paw-licking test, suggesting that **DHHPD** induces analgesia possibly by modulating the glutamatergic system through activation of the ionotropic and/or metabotropic glutamate receptors, and the suppression of NO production. 

Lastly, intraperitoneal administration of **DHHPD** at all doses for seven consecutive days did not induce any mortality, morbidity, behavioural changes or apparent gross post-mortem lesions in the vital organs of mice. These results suggest that, in addition to the protective effects seen in all models of nociception evaluated, **DHHPD** did not appear to impair the function of vital organs macroscopically and at this point considered safe for use.

## 4. Materials and Methods

### 4.1. Synthesis of **DHHPD**

**DHHPD** was synthesized in the Laboratory of Natural Products, Institute of Bioscience, Universiti Putra Malaysia (UPM), Serdang, Malaysia by Dr Leong Sze Wei and associates. The synthesis process of **DHHPD** involved a series of reactions including Knoevenagel reaction, phenolic esterification, Baker-Venkataraman rearrangements, and demethylation [28]. Briefly, a mixture containing 3,4-dimethoxybenzaldehyde, malonic acid and piperidine in pyridine underwent a reflux process to produce a mixture that was poured unto a flask containing cold diluted hydrochloric acid (HCl). The resulting precipitate was filtered and washed to afford mixture I. Following this process, phosphoryl chloride (POCl_3_) was added to mixture I and 2-hydroxyacetophenone in pyridine. The reaction mixture was then poured into cold diluted HCl, followed by extraction with ethyl acetate (EA). The organic layer was dried over the anhydrous magnesium sulphate and concentrated in vacuo before purification by open column chromatography to produce mixture II.

Next, ground potassium hydroxide pellets were added to a flask containing mixture II in pyridine. This mixture was poured into cold diluted HCl and then dried over anhydrous magnesium sulphate and evaporated under vacuo. Then, the solvent was purified with open column chromatography creating mixture III.

**Figure 7:**
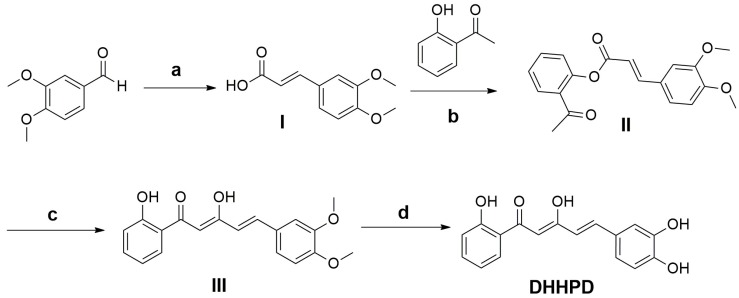
Synthesis of **DHHPD**. *Reagents and Conditions*: (**a**) malonic acid, pyridine, reflux process (4 h); (**b**) POCl_3_, pyridine, RT (overnight); (**c**) KOH, pyridine, RT (overnight); (**d**) BBr_3_, CH_2_Cl_2_, 0 °C (8 h). Reaction scheme and description adapted from [28].

Boron tribromide (BBr_3_) was added to mixture III in dry dichloromethane at freezing point. Then, mixture III was extracted with EA and dried over anhydrous magnesium sulphate to produce an organic layer which was dried in vacuo using a rotary evaporator (Rotavapor® R-300 System, Heidolph Instruments GmbH & CO. KG, Germany) before it underwent a final purification process by open column chromatography (Merck silica gel 60, mesh 70–230 and elution with 95% chloroform:5% methanol) to produce **DHHPD** (Figure 7). The structure and the purity of the compound were identified and characterized by using ^1^H-NMR and ^13^C-NMR (Varian 500 MHz, Varian Inc., Palo Alto, California, USA), HPLC utilizing Waters Xbridge C18 column (5 µm, 150 mm × 4.6 mm)(Thermo Finnigan Surveyor, San Josè, CA, USA) and gas chromatography mass spectrometry (Shimadzu GCMS-QP5050A Mass Spectrometer, Shimadzu, Kyoto, Japan). Colour: Orange; Yield: 37.15%; m.p.: 155–156 °C; Mass calculated: 298.0841; Mass found: 298.0843. ^1^H-NMR (500 MHz, acetone-d_6_) δ ppm 6.65–6.73 (m, 2H) 6.88–7.00 (m, 3H) 7.11 (d, *J* = 8.2 Hz, 1H) 7.23 (s, 1H) 7.52 (t, *J* = 7.86 Hz, 1H) 7.61 (d, *J* = 15.73 Hz, 1H) 7.92 (d, *J* = 8.15 Hz, 1H) 8.28 (br. s, 1H) 8.61 (br. s, 1H) 12.19 (s, 1H) 14.81 (br. s., 1H). ^13^C-NMR (126 MHz, acetone) δ ppm 96.4, 114.3, 115.6, 118.3, 119.0, 119.1, 119.1, 121.9, 127.5, 128.9, 135.8, 140.6, 145.6, 148.0, 162.4, 176.2, 195.6. A detailed description of the general synthetic steps for **DHHPD** was given in [28]. 

### 4.2. Chemicals and Drugs

The chemicals used are absolute alcohol, dimethyl sulfoxide (DMSO), Tween 20, 0.8% acetic acid, acetylsalicylic acid, morphine hydrochloride, 2.5% formalin, naloxone hydrochloride, l-glutamic acid hydrochloride, capsaicin and capsazepine. All chemicals and drugs were bought from Sigma Aldrich (M) Sdn. Bhd., Kuala Lumpur (Malaysia) except for formalin (HMBG Chemicals, Progressive Scientific Sdn. Bhd., Selangor, Malaysia). **DHHPD** was dissolved in vehicle consisting of 5% DMSO, 5% Tween 20 and 90% distilled water. The final concentration of DMSO and Tween 20 did not surpass 10% and without apparent negative effect *per se*. Chemical solutions were freshly prepared prior to the experiment and were administered through intraperitoneal (i.p.) injection at a volume of 10 mL/kg.

### 4.3. Animals

Male ICR mice (3–4 weeks, 20–30 g) were used throughout the experiments. Prior to the experiments, animals were acclimatized for 7 days in the Animal House, Faculty of Medicine and Health Sciences, UPM. Mice were used only once throughout the study and were immediately euthanized by cervical dislocation at the end of each experiment. Data was gathered in a blinded, randomized and controlled design for all experiments. This project has been approved by the Institutional Animal Care and Use Committee, UPM (Reference: UPM/IACUC/AUP-R088/2017).

### 4.4. Acute Toxicity Test

Acute toxicity test was performed according to [56], with slight modifications. Mice (6 per group) were fasted overnight and were given **DHHPD** (0.1, 0.3, 1 and 3 mg/kg, i.p.) and vehicle (10 mL/kg, i.p.) on the following day. Observation for occurrence of abnormal behaviours or any incidence of mortality was performed daily for 7 consecutive days. On day 8, mice from each group were euthanized by cervical dislocation and post mortem was conducted.

### 4.5. Antinociceptive Studies

#### 4.5.1. Acetic Acid-Induced Writhing Test

The 0.8% acetic acid-induced writhing test was conducted according to [57]. Mice (6 per group) were administered with vehicle (10 mL/kg, i.p.), acetylsalicylic acid (ASA) (100 mg/kg, i.p.) and **DHHPD** (0.1, 0.3, 1, 3 mg/kg, i.p.) 30 min prior to the administration of 0.8% acetic acid at the volume of 10 mL/kg (i.p.). The cumulative number of writhes exhibited by each animal was recorded beginning 5 min after the acetic acid injection for a period of 30 min.

#### 4.5.2. Formalin-Induced Paw Licking Test

The 2.5% formalin-induced paw licking test was conducted based on [58]. Thirty minutes before the intraplantar administration of 20 µL of 2.5% formalin, mice (6 per group) were administered with vehicle (10 mL/kg, i.p.), ASA (100 mg/kg, i.p.), morphine (5 mg/kg, s.c.) and **DHHPD** (0.1, 0.3, 1, and 3 mg/kg, i.p.). Each mouse was placed into the Perspex observation chamber immediately and the duration that each mouse spent licking and biting the injected paw at 0–5 min (early/neurogenic phase) and 15–30 min (late/inflammatory phase) post injection was recorded. 

#### 4.5.3. Hot-Plate Test

This test was performed according to [40] with slight modifications. Mice were preselected 24 h before the hot-plate test. Possible variation of results was minimized by selecting only mice with a baseline response latency of six to eight seconds. Mice with a baseline latency of less than 4 s were considered to be oversensitive and were excluded. Next, mice (*n* = 6) were administered with vehicle (10 mg/kg, i.p.), morphine (5 mg/kg, s.c.) and **DHHPD** (0.1, 0.3, 1, 3 mg/kg, i.p.), 30 min prior to the hot-plate test. The hot-plate (Model PE34, Series 8, IITC Inc. Life Science, Victory Blvd Woodland Hills, California, USA) was maintained at 52.5 ± 0.5 °C throughout the experiment. Following pre-treatment, mice were immediately placed on the hot-plate once every 30 min during the 240 min test period. The response latency recorded was the time taken between the placement of mice on the hot-plate and the display of either shaking, licking of paws or jumping response. The cut-off time set for each mouse on the hot-plate was 20 s to avoid paw tissue injury.

#### 4.5.4. Involvement of the Opioidergic System

A separate group of mice (*n* = 6) received naloxone (5 mg/kg, i.p), a non-selective opioid receptor antagonist, 15 min prior to the injection of **DHHPD** (3 mg/kg, i.p.). The subsequent procedure and evaluation for the hot-plate test were repeated.

#### 4.5.5. Capsaicin-Induced Paw Licking Test

The capsaicin-induced paw licking test was conducted according to [42]. Mice (6 per group) received vehicle (10 mL/kg, i.p.), capsazepine (0.17 mmol/kg, i.p.) and **DHHPD** (0.1, 0.3, 1, 3 mg/kg, i.p.). Thirty minutes post treatment, each mouse was injected with capsaicin (1.6 µg/paw, 20 µL, i.pl.) on its left hind paw. Mice were placed in a cylindrical Perspex observation chamber immediately and were observed for 5 min. The duration that each mouse spent on licking the injected paw was recorded as an indicator of nociception.

#### 4.5.6. Glutamate-Induced Paw Licking Test

This test was conducted according to [59]. Mice (*n* = 6) were administered with vehicle (10 mL/kg, i.p.), ASA (100 mg/kg, i.p.) and **DHHPD** (0.1, 0.3, 1, 3 mg/kg, i.p.). Each mouse was injected with a glutamate solution (10 µmol/paw, 20 µL, i.pl) on its left hind paw, 30 min after the pre-treatment. Mice were transferred into a cylindrical Perspex observation chamber immediately and were observed for 15 min. The duration that each mouse spent on licking and biting its injected paw was recorded. 

### 4.6. Motor Performance Test

#### Rotarod Test

The rotarod test was performed according to [60], with slight modification. Mice were pre-selected 24 h prior to the rotarod test. Mice that were capable of remaining on the revolving bar of the rotarod (Ugo-Basile, model 47600, Gemonio (VA), Italy) at 20 rpm for two consecutive periods of 60s without failing were selected. Following pre-selection, mice (6 per group) were administered with vehicle (10 mL/kg, i.p.), **DHHPD** (0.1, 0.3, 1 and 3 mg/kg, i.p.) and diazepam (4 mg/kg, i.p.), 30 min prior to the test. Following pre-treatment, mice were placed on the revolving bar of the rotarod (20 rpm) and their motor performance was assessed at 30, 60 and 90 min. The length of time each mouse remained on the revolving bar was recorded. 

### 4.7. Statistical Analysis

Statistical differences between groups were analysed using analysis of variance (ANOVA) with *p* < 0.05 as the limit of significance with Dunnett’s test as the post hoc test. Results from the hot-plate test were analysed by a two-way ANOVA followed by the Bonferonni post hoc test. The ED_50_ value (effective dose producing 50% inhibition in accordance to control value) was determined by nonlinear regression analysis using GraphPad Prism 6 (GraphPad Software Inc., San Diego, CA, USA). Results for all experiments are expressed as mean ± SEM.

## 5. Conclusions

In conclusion, **DHHPD** (1 and 3 mg/kg) showed excellent inhibition of nocifensive behaviour as shown in the acetic acid-induced writhing test, formalin-induced paw licking test and the hot-plate test. The peripheral and central antinociception may be mediated by modulation of the vanilloid pathway and glutamatergic system but does not involve the participation of opioidergic system. Further research on the effect of **DHHPD** in inhibiting the synthesis/release of inflammatory mediators by means of in vivo animal models of inflammation is recommended.

## Figures and Tables

**Table 1:** Effect of **DHHPD** against the hot-plate test in mice.

		Interval Following Treatment (min)								
Treatment		0	30	60	90	120	150	180	210	240
	Dose (mg/kg)	Latency Time (s)								
Vehicle (10 mL/kg)	-	6.16 ± 0.17	6.83 ± 0.30	6.67 ± 0.33	6.67 ± 0.21	6.83 ± 0.31	6.67 ± 0.21	6.83 ± 0.31	6.33 ± 0.21	6.30 ± 0.03
**DHHPD**	0.1	7.08 ± 0.35	7.94 ± 1.11	8.11 ± 0.56	9.58 ± 1.31 **	8.79 ± 0.45	7.29 ± 0.54	7.08 ± 0.36	6.57 ± 0.59	8.17 ± 0.84
	0.3	7.34 ± 0.34	8.30 ± 0.93	9.28 ± 0.34 *	10.01 ± 0.72 **	9.62 ± 0.80 *	8.83 ± 0.38	7.92 ± 0.44	8.21 ± 0.48	8.51 ± 0.63
	1	6.52 ± 0.23	7.58 ± 1.10	11.80 ± 1.22 ****	10.14 ± 1.34 ***	9.67 ± 0.42 *	10.60 ± 0.50 ****	10.80 ± 1.14 ****	7.59 ± 0.48	8.30 ± 0.90
	3	7.14 ± 0.39	9.51 ± 0.43 *	10.93 ± 0.78 ****	11.55 ± 1.06 ****	12.34 ± 1.07 ****	10.23 ± 0.45 ***	8.84 ± 0.53	9.41 ± 0.61 **	7.68 ± 0.34
Morphine	5	7.50 ± 0.34	18.33 ± 0.67 ****	17.00 ± 0.82 ****	16.33 ± 0.42 ****	16.33 ± 0.99 ****	15.33 ± 0.62 ****	15.17 ± 0.40 ****	14.83 ± 0.40 ****	13.70 ± 0.05
Naloxone + **DHHPD**	5 + 3	7.13 ± 0.22	08.56 ± 0.52	9.79 ± 1.05	9.01 ± 0.89	13.76 ± 1.03	9.95 ± 0.43	8.25 ± 0.71	7.30 ± 0.53	7.88 ± 0.35
Naloxone + Morphine	5 + 5	6.33 ± 0.21	6.67 ± 0.33 ^#^	6.67 ± 0.33 ^#^	6.50 ± 0.22 ^#^	7.00 ± 0.37 ^#^	6.50 ± 0.22 ^#^	6.83 ± 0.17 ^#^	6.50 ± 0.22 ^#^	6.36 ± 0.01 ^#^

Results are presented as mean ± SEM of latency time (s) of six mice. The asterisks (*) represent the significance level compared to control, * *p* < 0.5, ** *p* < 0.01, *** *p* < 0.001, **** *p* < 0.0001 by two-way ANOVA followed by Bonferroni’s post hoc test. The hash (#) denotes the significance level, ^#^ *p* < 0.0001 compared to the morphine group.
